# Prefrontal dysfunction in post-COVID-19 hyposmia: an EEG/fNIRS study

**DOI:** 10.3389/fnhum.2023.1240831

**Published:** 2023-09-27

**Authors:** Livio Clemente, Marianna La Rocca, Nicola Quaranta, Lucia Iannuzzi, Eleonora Vecchio, Antonio Brunetti, Eleonora Gentile, Michele Dibattista, Simona Lobasso, Vitoantonio Bevilacqua, Sebastiano Stramaglia, Marina de Tommaso

**Affiliations:** ^1^Department of Translational Biomedicine and Neuroscience (DiBraiN), University of Bari Aldo Moro, Bari, Italy; ^2^M. Merlin Inter-university Physics Department, University of Bari, Bari, Italy; ^3^Laboratory of Neuroimaging, Keck School of Medicine of USC, USC Stevens Neuroimaging and Informatics Institute, University of Southern California, Los Angeles, CA, United States; ^4^Department of Electrical and Information Engineering, Polytechnic University of Bari, Bari, Italy

**Keywords:** long COVID-19, anosmia, near infra-red spectroscopy, event related potentials, cognitive dysfunction

## Abstract

**Introduction:**

Subtle cognitive dysfunction and mental fatigue are frequent after severe acute respiratory syndrome coronavirus 2 (SARS-CoV-2) infection, characterizing the so-called long COVID-19 syndrome. This study aimed to correlate cognitive, neurophysiological, and olfactory function in a group of subjects who experienced acute SARS-CoV-2 infection with persistent hyposmia at least 12 weeks before the observation.

**Methods:**

For each participant (32 post-COVID-19 patients and 16 controls), electroencephalography (EEG) and functional near-infrared spectroscopy (fNIRS) data were acquired using an integrated EEG–fNIRS system during the execution of a P300 odd-ball task and a Stroop test. The Sniffin' Sticks test was conducted to assess subjects' olfactory performance. The Montreal Cognitive Assessment (MoCA) and the Frontal Assessment Battery (FAB) were also administered.

**Results:**

The post-COVID-19 group consisted of 32 individuals (20 women and 12 men) with an average education level of 12.9 ± 3.12 years, while the control group consisted of 16 individuals (10 women and 6 men) with an average education level of 14.9 ± 3.2 years. There were no significant differences in gender (*X*^2^ = 0, *p* = 1) or age between the two groups (age 44.81 ± 13.9 vs. 36.62 ± 11.4, *p* = 0.058). We identified a lower concentration of oxyhemoglobin (*p* < 0.05) at the prefrontal cortical level in post-COVID-19 subjects during the execution of the Stroop task, as well as a reduction in the amplitude of the P3a response. Moreover, we found that post-COVID-19 subjects performed worst at the MoCA screening test (*p* = 0.001), Sniffin's Sticks test (*p* < 0.001), and Stroop task response latency test (*p* < 0.001).

**Conclusions:**

This study showed that post-COVID-19 patients with persistent hyposmia present mild deficits in prefrontal function, even 4 months after the end of the infection. These deficits, although subtle, could have long-term implications for quality of life and cognitive wellbeing. It is essential to continue monitoring and evaluating these patients to better understand the extent and duration of cognitive impairments associated with long COVID-19.

## 1. Introduction

Severe acute respiratory syndrome coronavirus 2 (SARS-CoV-2), the cause of the aggressively developing coronavirus infection outbreak (COVID-19) (Li et al., 2020), has rapidly spread worldwide (Bedford et al., 2020), and on March 11, 2020, the WHO declared COVID-19 as a pandemic (WHO, 2020). This incident could be seen as the start of a worldwide pandemic that, in just 2 months, has caused catastrophic devastation all across the world.

SARS-CoV-2 is an infection with a wide range of clinical manifestations. Nearly 40–60% of patients develop a loss of sense of smell, and many of them continue to complain of persistent symptoms, most of which are neurological or cognitive (Chaumont et al., 2020), up to 12 weeks following COVID-19 diagnosis (Ceban et al., 2022). Most worrisome are the long-term complications of the viral infection, and patients with previous SARS-CoV-2 infection may have residual olfactory, gustatory, and prefrontal and limbic lobe functional alterations (Najt et al., 2021). In COVID-19 patients, cognitive problems are often discovered months after hospital discharge, including memory loss and slowed cognitive processing speed that may affect patients' everyday life (Ferrucci et al., 2021).

The post-COVID-19 syndrome is defined by the National Institute for Health and Care Excellence (NICE) as a set of signs and symptoms that arise during or after an infection consistent with COVID-19 and cannot be explained by another diagnosis. It is characterized by clusters of symptoms that frequently coexist and can fluctuate over time, affecting every system of the body (World Health Organization, 2021). In the first model, Fernández-de-las-Peñas et al. (2021) divided the acute phase of SARS-CoV-2 infection into four stages, each corresponding to a specific post-COVID-19 symptom: (1) possibly infection-related symptoms that last up to 4–5 weeks after they first appear; (2) acute post-COVID-19 symptoms that last from week 5 to 12 after symptoms first appear; (3) protracted post-COVID-19 symptoms that last from week 12 to 24; and (4) persistent post-COVID-19 symptoms, lasting more than 24 weeks after they first appear.

Fatigue, shortness of breath, coughing, joint discomfort, chest pain, muscle pain, headache, and other symptoms are some of the typical post-COVID-19 symptoms. In addition, Davis et al. (2021) found that people also reported post-exertional malaise and cognitive dysfunction.

In a systematic review and meta-analysis, Ceban et al. (2022) aimed to quantify the proportion of individuals who experience fatigue and cognitive impairment 12 or more weeks after the diagnosis of COVID-19 and to characterize the inflammatory correlates and functional consequences of the syndrome. They found that approximately a third of the included individuals experienced persistent fatigue, and over one-fifth of individuals exhibited cognitive impairment following the COVID-19 diagnosis. Neurocognitive symptoms worsen after 22 weeks, while improvements occur in most other symptoms (Jason et al., 2021).

Recent research on the spatial distribution of cortical and subcortical activation during cognitive tasks has generated considerable interest in the simultaneous recording of event-related potentials (ERP) and the related functional near-infrared spectroscopy (fNIRS) response (Jaquerod et al., 2021). This method could be focalized on the frontal regions, which could be easily aggressed by the direct viral entry through the nasal mucosa (Meinhardt et al., 2021). Sato et al. (2013) recently investigated prefrontal cortex (PFC) activity in healthy controls using simultaneous NIRS-fMRI measurements. They found that prefrontal NIRS-Hb signals exhibited a significant correlation with blood oxygen level-dependent (BOLD) signals in the activation area. This finding, combined with the simultaneous use of EEG-fNIRS for performing accurate mental workload classification (Liu et al., 2017), led us to employ an integrated EEG-fNIRS recording system in our study.

We hypothesize that patients with previous SARS-CoV-2 infection experiencing protracted smell impairment might have residual prefrontal functional alterations; therefore, the purpose of our study was to evaluate functional brain analysis and frontal cognitive performance in subjects with mild protracted hyposmia compared to subjects with no previous COVID-19 infection.

## 2. Materials and methods

### 2.1. Subjects

A total of 48 subjects were recruited at the Neurophysiopathology Unit of Bari Policlinico General Hospital, Italy to participate in the study, including 32 patients with a confirmed diagnosis of SARS-CoV-2 infection who had recovered at least 3 months prior to the study and with mild symptoms of hyposmia. All patients reported mild acute symptoms, such as fever, myalgia, flu, mild dyspnea, and headache, which did not require hospitalization. In all cases, acute hyposmia and hypogeusia were experienced. In all, 16 healthy controls, with no history of SARS-CoV-2 infection and asymptomatic at the time of evaluation, were also examined. The study was performed from January 2021 to January 2022. All subjects were right-handed and above 18 years of age. It is worth noting that for the purpose of this study, only non-smokers or those who had stopped smoking for at least 1 year were included (Murphy, 2002). The experimental procedures were approved by the ethics committee of the General Polyclinic of Bari and performed in accordance with the Declaration of Helsinki. Prior to the experiment, all subjects received explanations about the study's goals and gave their written informed consent. All the participants in this study were able to independently follow the study instructions and had no prior knowledge of the recording devices or the experimental task.

Other factors, identifiable as exclusion criteria, were considered: (a) severe and untreatable medical conditions (heart, lung, kidney, and liver failure), ongoing neoplastic diseases, present or past central nervous system (CNS) diseases unrelated to COVID-19; (b) known pathology or past rhino sinus surgery; (c) active or remote use of inhaled volatile substances or nasal topical vasoconstrictors; (d) head injury; (e) use of drugs that affect the CNS in the week preceding the study; and (f) psychiatric disorders, according to DSM-5.

### 2.2. Experimental paradigm

**Figure 1:**
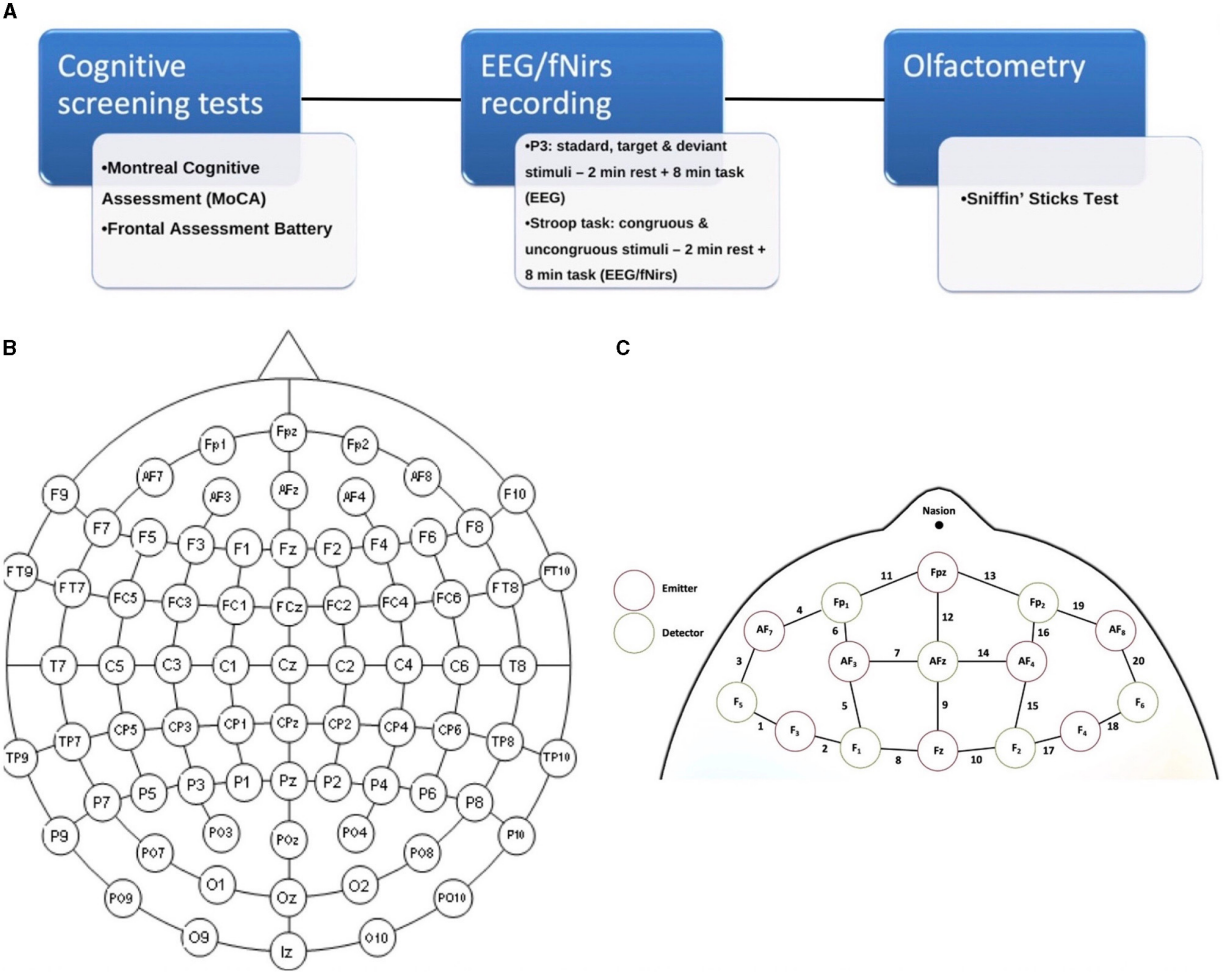
**(A)** Experimental paradigm of the study; **(B)** 61 channels EEG-enlarged international 10–20 system; and **(C)** fNIRS prefrontal region, including the distinction between emitters and detectors.

First, subjects underwent the cognitive screening assessment (Figure 1A); thereafter, they were examined with the integrated EEG/fNIRS cap while sitting in a comfortable chair in a well-ventilated room and positioned in front of a screen. The tasks administered to study participants were the Stroop test (Caffarra et al., 2002) and a P3 paradigm, including target and non-target deviant stimuli (Polich, 2007) (see below). Both tasks involved an initial 2-min resting state baseline in which the subject was asked to stare at a cross in the center of the black screen. Finally, subjects were accompanied to the otorhinolaryngology operative unit for an evaluation of the degree of hyposmia by means of olfactometry (Sniffin' Sticks test).

Cognitive screening assessment: The participants received the Montreal Cognitive Assessment (MoCA) (Pirrotta et al., 2015) and the Frontal Assessment Battery (FAB) (Appollonio et al., 2005). The computerized version of the Stroop test—abbreviated version-was used (Caffarra et al., 2002). Interference test: Name the color of ink used to write the word in the shortest possible time, was also triggered with the EEG/fNIRS recording.

The Sniffin' Sticks test (Hummel et al., 1997) is an instrument for assessing nasal chemosensory abilities using pen-like odor delivery devices. The pens were ~14 cm long and had an inner diameter of 1.3 cm; they were loaded with odorants that were liquid or dissolved in propylene glycol, with a total volume of 4 ml. To administer the odor to be evaluated, the investigator removed the cap for ~3 s and positioned the tip of the pen ~2 cm from both nostrils.

Analysis of dependent variables was performed using Student's *t*-test for unpaired data (2-tailed) comparing the neuropsychological scores relative to the two groups (“patients” vs. “controls”). Differences were considered statistically significant by setting a *p*-value of < 0.05.

#### 2.2.1. Recording technique

##### 2.2.1.1. fNIRS-EEG co-recording

The cerebral hemodynamic and bioelectrical activity was recorded using an EEG-fNIRS co-recording cap with an additional black cap placed over it to block out any potential ambient light interference. The fNIRS device is a multi-channel system able to measure hemodynamic activity variations. For data acquisition, the researchers adopted NIRStar 14.2 software (Version 14, Revision 2, Release Build, 2016-04-15 NIRx Medizintechnik GmbH, Berlin, Germany; www.nirx.net). The easy-to-use device involved LED sources and photosensitive detectors (sensitivity: >1 pW, dynamic range: >50 dB). Two LEDs were used in each source; each of them generated near-infrared light with a wavelength of 760 and 850 nm and a sampling rate of 7.81 Hz. For EEG recording, a Micromed Brain Quick apparatus was used. The EEG/fNIRS cap consisted of 61 encephalic EEG channels positioned according to the enlarged international 10–20 system (Fp1, Fpz, Fp2, F7, F3, Fz, F4, F8, T3, C3, Cz, C4, T4, T5, P3, Pz, P4, T6, O1, Oz, O2, AF7, AF3, AFz, AF4, AF8, F5, F1, F2, F6, FT7, FC5, FC3, FC1, FCz, FC2, FC4, FC6, FT8, C5, C1, C2, C6, TP7, CP5, CP3, CP1, CPz, CP2, CP4, CP6, TP8, P5, P1, P2, P6, PO7, PO3, POz, PO4, and PO8). A biauricular reference electrode was used. Eight light sources and eight detectors were positioned in the prefrontal region according to a predefined montage (Figures 1B, C). In addition, two electrooculogram detection electrodes were applied to remove any ocular blink artifacts placed at the level of the outer canthus of the right and left eye, while the ground electrode was placed on the right forearm. The impedance was kept below 5 KΩ. During the EEG recording, we used a digital filter in the 0.1–70 Hz range and a 50 Hz notch filter to allow signal inspection.

There were a total of 20 fNIRS measurement channels given by the combinations of sources and detectors, with 10 channels in each hemisphere's region.

#### 2.2.2. Event-related potentials paradigms

##### 2.2.2.1. Visual P3

The frequency of occurrence of the standard stimulus (big sphere) of the P3 task was 80%, while for the target stimulus (small sphere) and the deviant stimulus (cylinder), it was 10%, respectively. A total of 250 stimuli were administered, with a duration of 0.5 s and an inter-stimulus interval time of 2 s. Participants were instructed to press the spacebar whenever the target stimulus appeared in the shortest possible time.

##### 2.2.2.2. Stroop task

Stimuli were displayed individually on a monitor with a black background. During the test, a list of words (green, blue, and red) was presented in a randomized sequence; however, the ink color of the words could be concordant or discordant with the word presented (e.g., the word “blue” shown in blue ink or the word “blue” shown in green ink). Then, subjects were asked to consider the color of the ink and ignore the semantic meaning of the word (e.g., to answer “green” instead of “blue”, considering the case of the previous example) by quickly pressing the space key. The total stimuli administered were 60 and were presented on the screen for 2 s, with an interstimulus interval time of 5 s. The stimuli consisted of the words blue, red, and green and were presented in randomized order as follows: 10 congruent “blue” (“blue” word colored blue); 10 congruent “red”; 10 congruent “green”; 10 incongruent “blue” (5 colored red and 5 green); 10 incongruent “reds” (5 colored blue and 5 green); and 10 incongruent “green” (5 colored red and 5 blue).

### 2.3. Data analysis

#### 2.3.1. EEG data processing

The EEG data were processed with an automatic pipeline based on EEGLAB (v2022) running on MATLAB. The data were filtered between 1 and 30 Hz using an FIR filter. Then, we used the artifact subspace reconstruction method to correct continuous data and reject bad channels and data segments. Then, we interpolated the bad channels and re-referenced all the data to the average. The maximum acceptable standard deviation for a window of 0.5 s was considered to be 20. In addition, we removed channels that were flat for more than 5 s, channels with a standard deviation of high-frequency noise <4, and channels that had a correlation with neighboring channels >0.8.

Next, an independent component analysis was carried out, and artifact components were automatically rejected by using a machine learning algorithm called the Multiple Artifact Rejection Algorithm. Components with a probability of being artifacts higher than 0.50 were removed. The data were then epoched in the time interval −0.1 to 1 s, and the baseline was corrected.

ERP analyses of EEG signals were conducted for both the P3 odd-ball paradigm and the Stroop test using the MATLAB-based Letswave 7 tool. For the P3 oddball paradigm, the data were categorized into three distinct conditions: standard, target, and deviant non-target stimuli. The epochs were averaged for each participant, and an independent-samples *t*-test with non-parametric permutation analysis was carried out for each condition. The P3 response was the maximal positive response in the time frame of 250–450 ms.

The deviant non-target stimuli latency was computed at the frontocentral electrode site FCz, while target stimuli-related response latencies were measured at the parietal electrode site Pz (Polich, 2007).

For the Stroop test analysis, the data were partitioned into two conditions: congruent stimuli (e.g., the word “red” displayed in red ink) and incongruent stimuli (e.g., the word “red” presented in green ink). The maximal negative response was checked within the time interval of 450–500 ms (e.g., N400 effect) and in the 600–1,000 ms range for the late sustained potential (LSP). Latencies measured on the Fcz electrode were considered (Schack et al., 1999; Heidlmayr et al., 2020). Similar to the P3 oddball paradigm, the epochs were averaged for each condition in both groups, followed by a comparison using an independent-samples *t*-test with non-parametric permutation analysis, available within the Letswave MATLAB tool.

#### 2.3.2. fNIRS data processing

The fNIRS signal processing was carried out using nirsLAB (version 2017.6). First, discontinuities were eliminated before performing the signal processing. Then, we used the Remove Spike Artifacts GUI of nirsLAB to automatically identify and eliminate the two most frequent forms of artifacts found in fNIRS data: spike and baseline shifts. In order to remove low oscillations from the fNIRS signal, such as respiratory and cardiac frequencies, the raw data were filtered in the band-pass range of 0.008–0.2 Hz. The W. B. Gratzer method was used to convert the processed signals to optical intensities, and the optical intensities were then converted to changes in oxyhemoglobin and deoxyhemoglobin concentrations using a modified version of the Beer-Lambert law. Before calculating the changes in hemoglobin concentration, we performed a baseline adjustment, which was defined as the first 20 s of the 120 s of total resting state time that was recorded before each task. In the present study, we considered oxyhemoglobin levels.

We considered the changes in oxyhemoglobin during the performance of the Stroop test, considering the stimuli-related changes in a time window of 5 s after the stimulus appearance.

We performed topographical analysis and identified the brain regions that were active during the completion of the tasks in each individual case using the generalized linear model based on a Statistical Parameter Mapping NIRS-SPM (SPM 8) program (Ye et al., 2009), which was implemented in NIRSlab. We used the hemodynamic response function to represent the hemodynamic response to the experimental tasks in the statistical parametric mapping (SPM1-within subject) analysis, calculating the degree of activation on each channel relative to the baseline (beta value). Finally, the SPM2 (between participants) analysis was carried out to identify the fNIRS channels wherein HbO varied substantially in the Stroop task between groups using Student's *t*-test with *p* < 0.05, corrected for multiple comparisons.

In order to observe possible neuropsychological and neurophysiological correlates of hyposmia, we used the Pearson correlation test among the considered variables.

## 3. Results

The group of recovered COVID-19 patients did not differ significantly in gender (*X*^2^ = 0, *p* = 1) or age from the healthy control group (age 44.81 ± 13.9 vs. 36.62 ± 11.4, *p* = 0.058). The patient group consisted of 32 individuals (20 women and 12 men) with an average educational level of 12.9 ± 3.12 years. The control group, on the other hand, was made up of 16 individuals (10 women and 6 men) with an average educational level of 14.9 ± 3.2 years. Patients underwent cognitive screening tests and fNIRS measurements at an average of 20.6 weeks after recovery from COVID-19, with a range of 16.6–27.6 weeks.

### 3.1. Neuropsychological assessment and behavioral responses

Cognitive scores were compared across the groups (patients and controls), and a significant difference in the global MoCA score was found (Table 1). In detail, we observed a significant difference in the memory subgroup of the MoCA test. The other MoCA subgroups as well as the FAB test did not show any significant differences across the two groups.

**Table 1:** Mean, standard deviation, and statistical breakdown of neuropsychological tests, sniffing test, and main behavioral performances during ERPs paradigms.

	**Patients**	**Controls**	**Student's *t*-test**	***p*-value**
MoCa, mean (SD)	25.09 (±2.46)	27.62 (±2.18)	−3.474	0.001^***^
MoCa_visuospatial, mean (SD)	3.96 (±1.03)	4.31 (±0.47)	−1.262	0.213
MoCa_denomination, mean (SD)	2.96 (±0.17)	2.93 (±0.25)	0.501	0.619
MoCa_attention, mean (SD)	5.81 (±0.47)	5.68 (±0.87)	0.647	0.521
MoCa_language, mean (SD)	2.40 (±0.71)	2.75 (±0.44)	−1.760	0.085
MoCa_abstraction, mean (SD)	1.78 (±0.49)	2 (0)	−1.773	0.083
MoCa_memory, mean (SD)	2.62 (±1.33)	3.87 (±0.95)	−3.327	0.002^**^
MoCa_orientation, mean (SD)	5.87 (±0.33)	6 (0)	−1.480	0.146
Fab, mean (SD)	17.34 (±0.78)	17.56 (±1.09)	−0.795	0.431
Sniffin', mean (SD)	24.66 (±7.91)	33.45 (±1.21)	−4.394	< 0.001^***^
Stroop response latency (ms), mean (SD)	1,380.27 (±455.67)	979.07 (±175.66)	3.383	0.001^***^
Stroop_right, mean (SD)	55.65 (±5.62)	56.62 (±3.07)	0.640	0.525
Stroop_wrong, mean (SD)	2.62 (±3.26)	2.06 (±1.84)	0.639	0.526
Stroop_miss, mean (SD)	1.71 (±2.79)	1.31 (±1.66)	0.534	0.596
p3 response latency (ms), mean (SD)	557.25 (±122.25)	494.11 (±59.13)	1.948	0.058
p3_right, mean (SD)	22.5 (±3.3)	24.43 (±0.81)	−2.301	0.026^*^
p3_wrong, mean (SD)	1.28 (±1.63)	1.06 (±1.12)	0.481	0.633
p3_miss, mean (SD)	2.5 (±3.3)	0.56 (±0.81)	2.301	0.026^*^

* ≤ 0.05.

** ≤ 0.01.

*** ≤ 0.001.

In the data analysis, we observed that the response time to the Stroop test in the post-COVID-19 patients averaged 1,380.27 ± 455.67 ms, significantly longer than in the controls, who had an average response time of 979.07 ± 175.66 ms. This difference was confirmed by a *t*-test value of 3.383 and a *p*-value of 0.001 (Table 1).

Latencies of responses to the target stimuli during the P3 oddball paradigm were mildly but not significantly increased in post-COVID-19 subjects. The number of right responses was reduced and that of missing responses increased in participants with persistent post-COVID-19 hyposmia (Table 2).

**Table 2:** Descriptive of P3 features measured on the FCZ channel for the deviant stimulus and Pz channel for the target stimulus.

**Variables**	**Group**	***N***	**Mean**	***SD***	**Statistic**	***df***	***p*-value**
Target stimulus latency	Patients	32	0.256	0.0208	−1.147	46	0.257
	Controls	16	0.265	0.0294			
Deviant stimulus latency	Patients	32	0.305	0.0314	2.436	46	0.019^*^
	Controls	16	0.281	0.0329			
Target stimulus amplitude	Patients	32	2.542	1.2248	−1.491	46	0.143
	Controls	16	3.127	1.3913			
Deviant stimulus amplitude	Patients	32	2.880	1.4538	−2.857	46	0.006^**^
	Controls	16	4.252	1.7819			

H_a_ μ _1_ ≠ μ _2_ in Student's t-test.

* ≤ 0.05.

** ≤ 0.01.

#### 3.1.1. Sniffing test

As expected, we observed a significant reduction of smell capacities in patients with previous SARS-CoV-2 infection (Table 1).

### 3.2. EEG results

#### 3.2.1. P3 oddball paradigm

##### 3.2.1.1. Latencies

The latencies of the P3 component obtained from the target stimuli were similar between the patients and controls. Specifically, the target stimulus response latency was similar between patients and controls (Table 2). For deviant stimulus-related response latency, patients showed a significant delay (Table 2).

##### 3.2.1.2. Amplitudes

The amplitudes of the P3-related target stimuli did not significantly differ between the groups.

We observed a statistically significant difference between the two groups with regard to the amplitude of the P3 response after a deviant stimulus. In terms of latency, the mean value of the deviant stimulus for the patient group was 0.305 s with a standard deviation of 0.0314 s, while for the control group, it was 0.281 s with a standard deviation of 0.0329 s. In terms of amplitude, the mean value of the deviant stimulus for the patient group was 2.880 μV with a standard deviation of 1.4538 μV, while for the control group, it was 3.127 μV with a standard deviation of 1.3913 μV (Table 2).

**Figure 2:**
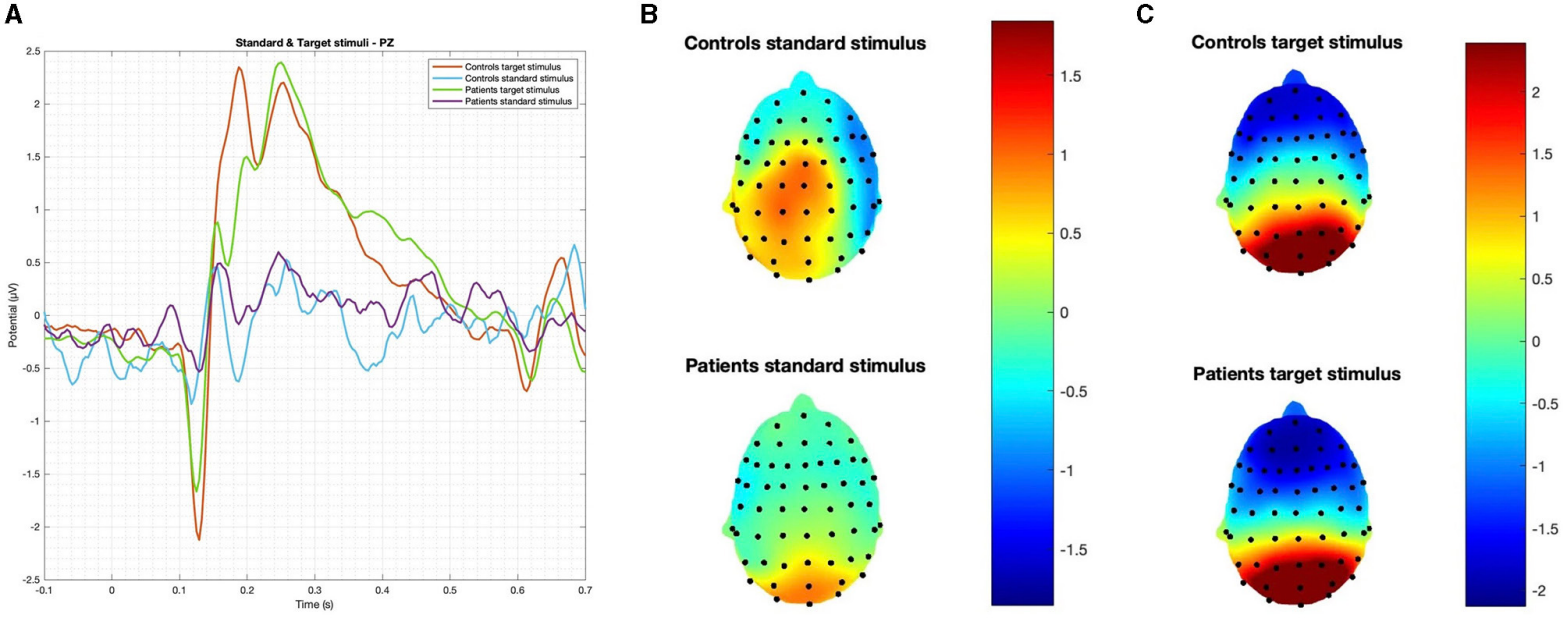
**(A)** Grand average on the Pz channel and topographic maps of evoked responses to **(B)** standard and **(C)** target stimuli. No significant difference was observed in latency and amplitude.

**Figure 3:**
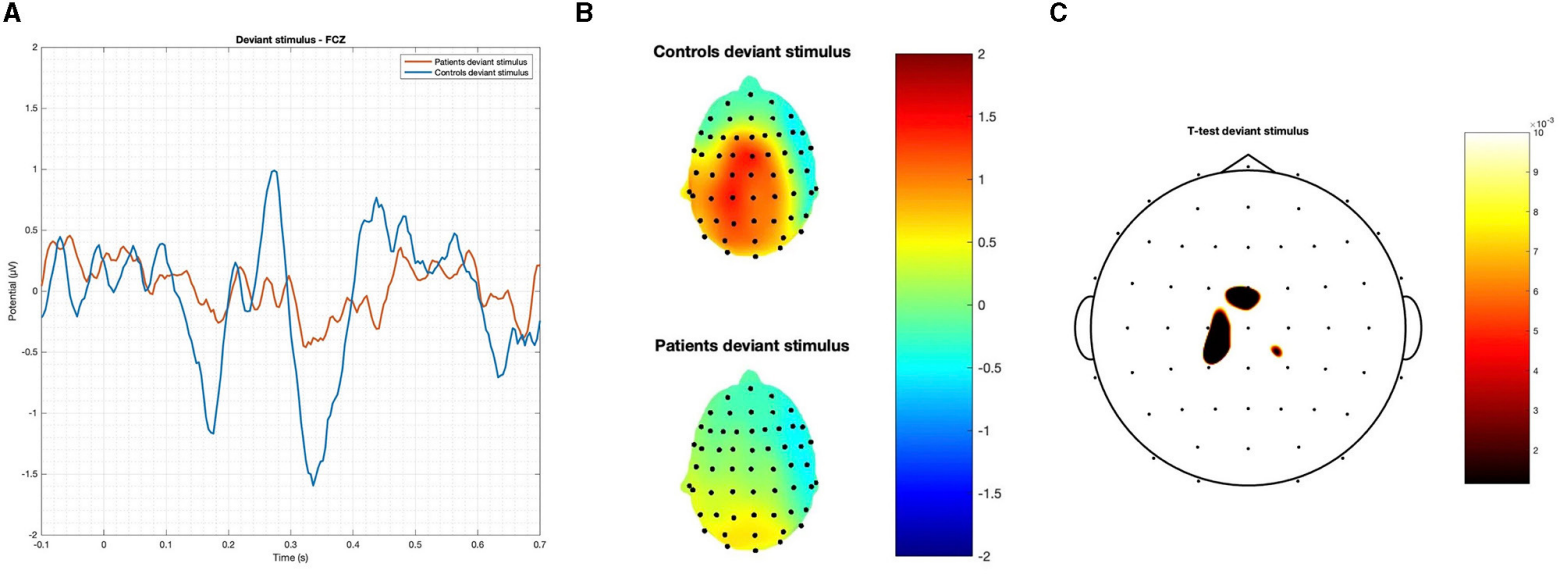
**(A)** Grand average on the FCz channel and **(B)** topographic maps of the P3 obtained by the deviant stimulus; and **(C)** *p*-values below 0.01 obtained with Student's *t*-test are reported.

In summary, while patients and controls showed similar responses to target stimuli, their responses to deviant stimuli were significantly different for both latency and amplitude (Figures 2, 3).

#### 3.2.2. Stroop task

**Figure 4:**
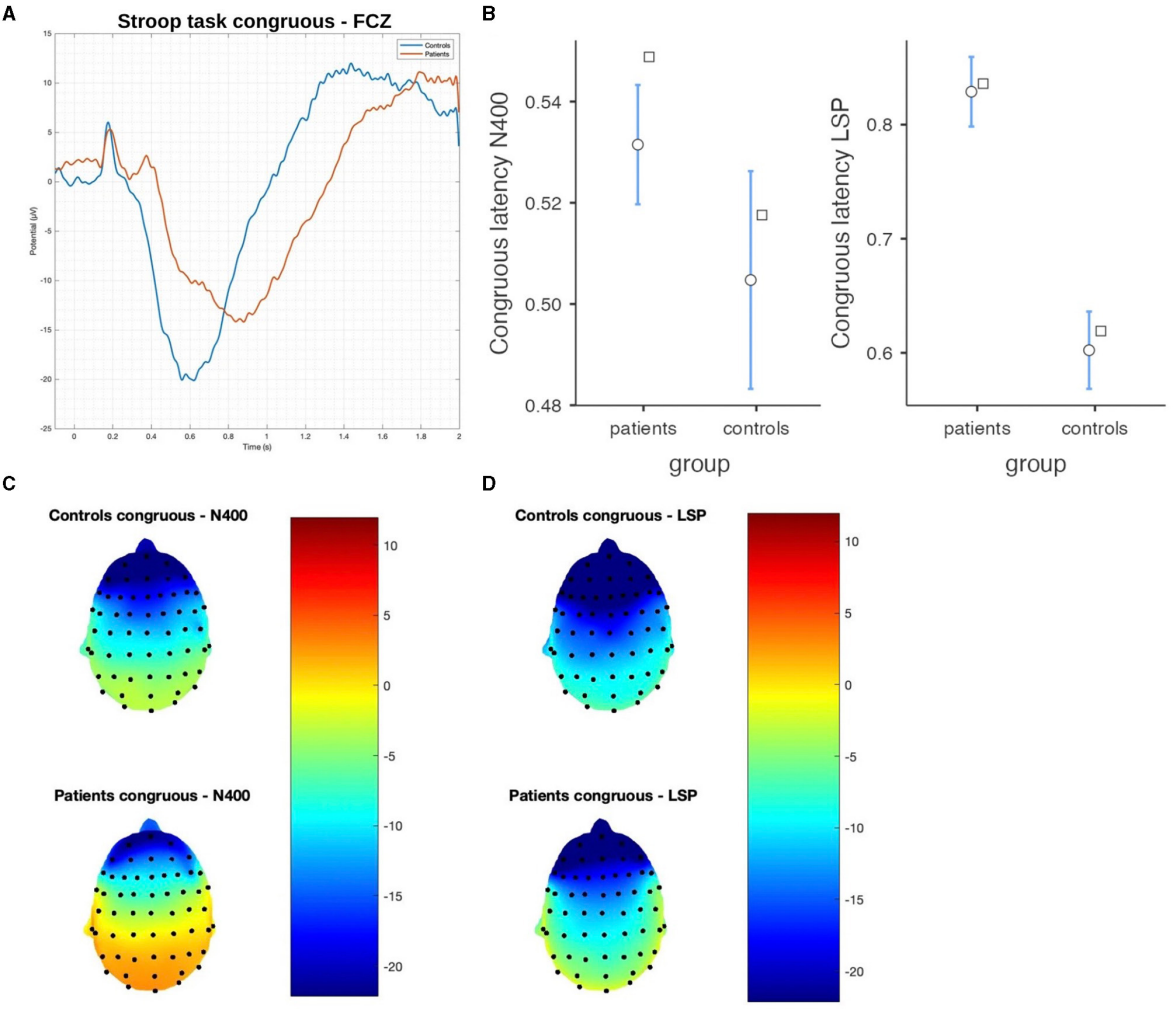
The difference between patients and controls during the Stroop test for the congruous stimulus: **(A)** ERP grand average; **(B)** descriptive plots of the N400 and LPS latencies measured on the FCZ channel (*p* < 0.01); **(C)** topographical maps for the N400 effect; and **(D)** topographical maps for the late sustained potential (LSP). No significant difference in amplitude was detected.

**Figure 5:**
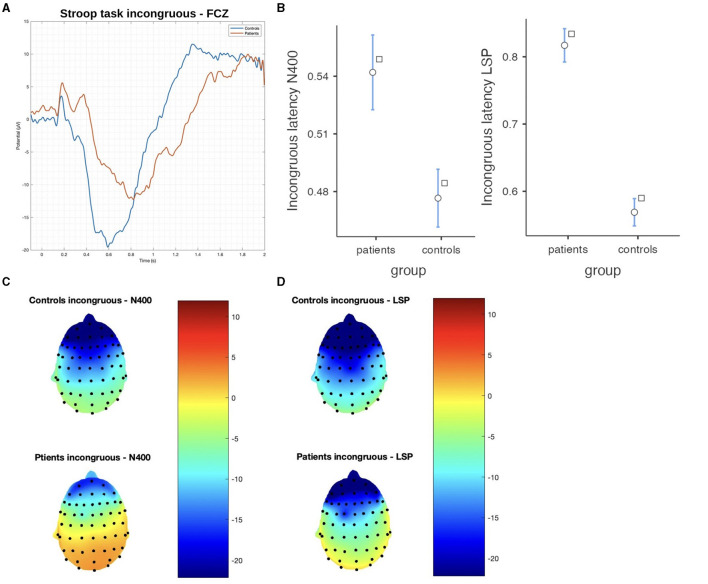
The difference between patients and controls during the Stroop test for the incongruous stimulus: **(A)** ERP grand average; **(B)** descriptive plots of the N400 and LPS latencies (*p* < 0.01) measured on the Fcz channel; **(C)** topographical maps for the N400 effect; and **(D)** topographical maps for the late sustained potential (LSP). No significant difference in amplitude was detected.

In the Stroop task, significant differences were observed between patients and controls in terms of N400 and LSP latencies, with patients demonstrating longer latencies in both congruous and incongruous conditions (*p* < 0.001). However, there were no significant group differences found in the N400 and LSP amplitudes, indicating similar ERP magnitudes between the groups (Table 3; Figures 4, 5). Moreover, the spatial distribution of both N400 and LSP showed reduced scalp diffusion of both negativities for the incongruent stimulus while approaching statistical significance.

**Table 3:** Descriptive of the Stroop task with numerosity of groups, mean, standard deviation (SD), and statistic across the groups.

**Variables**	**Group**	***N***	**Mean**	***SD***	**Statistic**	***df***	***p*-value**
Congruous latency N400	Patients	32	0.531	0.034	2.328	46	< 0.024^*^
	Controls	16	0.505	0.043			
Incongruous latency N400	Patients	32	0.542	0.0562	3.52	46	< 0.001^***^
	Controls	16	0.477	0.0307			
Congruous amplitude N400	Patients	32	−11.942	15.4582	1.55	46	0.133
	Controls	16	−20.188	21.365			
Incongruous amplitude N400	Patients	32	−11.943	14.9819	1.78	46	0.221
	Controls	16	−18.694	22.4716			
Congruous latency LSP	Patients	32	0.829	0.0881	10.31	46	< 0.001^***^
	Controls	16	0.602	0.0691			
Incongruous latency LSP	Patients	32	0.817	0.0715	5.6	46	< 0.001^***^
	Controls	16	0.569	0.0415			
Congruous amplitude LSP	Patients	32	−18.996	18.3857	1.21	46	0.469
	Controls	16	−23.240	20.2129			
Incongruous amplitude LSP	Patients	32	−15.773	20.2998	1.11	46	0.347
	Controls	16	−21.882	14.7814			

H_a_ μ _1_ ≠ μ _2_ in Student's t-test.

* ≤ 0.05.

*** ≤ 0.001.

### 3.3. fNIRS results

In the resting situation, we observed that patients had a tendency toward reduced cortical metabolism in the frontal regions, without statistical significance.

#### 3.3.1. Stroop test

**Figure 6:**
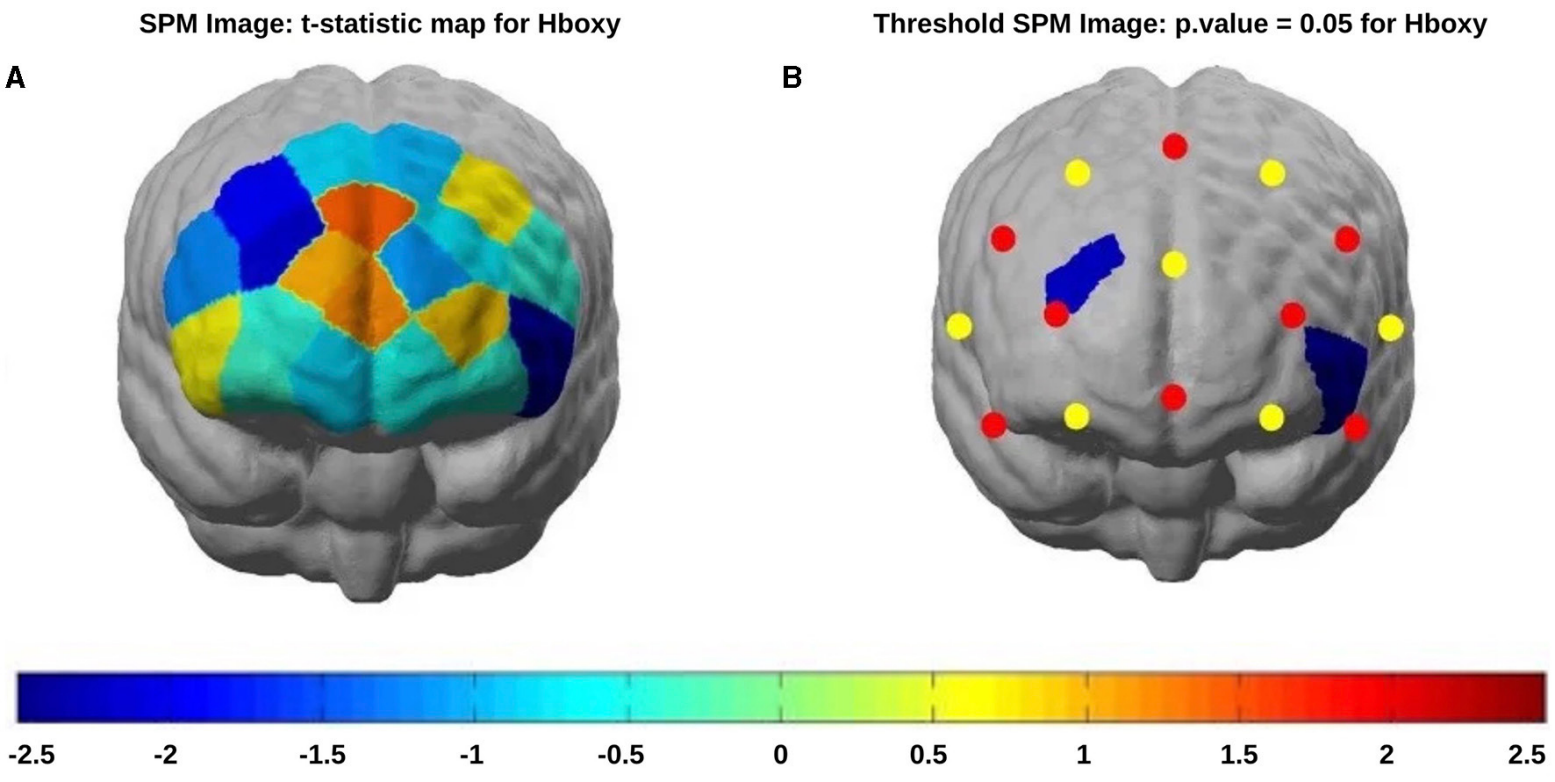
fNIRS *t*-statistic maps of the brain regions during the Stroop task incongruous stimulus showing the differences in the oxyhemoglobin comparisons of patients (red) vs. controls (blue): **(A)** Stroop task incongruous stimulus without threshold) and **(B)** Stroop task incongruous stimulus with threshold (*p* < 0.05).

In post-COVID-19 patients, a reduced HbO concentration emerged in channels 3 (*t*-statistic = −2.23, *p* = 0.032) and channel 14 after the incongruent stimuli appearance (*t*-statistic = −2.33, *p* = 0.023) (Figure 6). The levels of oxyhemoglobin recorded after the congruous stimulus appearance showed a tendency toward a reduction in post-COVID-19 patients without statistical significance.

#### 3.3.2. Correlations between hyposmia, neuropsychological, and neurophysiological features

The oxyhemoglobin levels showed a moderate positive correlation with the sniffing test results (Table 4); a substantial positive correlation was observed between “congruous stimulus latency LSP” and SST, indicating that as congruent latency at the LSP increases, so does the total sniff. A similar positive correlation was also noted between “incongruous stimulus latency LSP” and SST.

**Table 4:** Pearson values between the sniffing tests and hemodynamic and EEG correlates of the Stroop test.

**Variable**	**Pearson (*r*)**	***p*-value**
NIRS (oxyhemoglobin channel 3)	0.350	0.015^*^
Congruous stimulus latency LSP	0.466	< 0.001^***^
Incongruous stimulus latency LSP	0.438	0.002^**^

* ≤ 0.05.

** ≤ 0.01.

*** ≤ 0.001.

**Figure 7:**
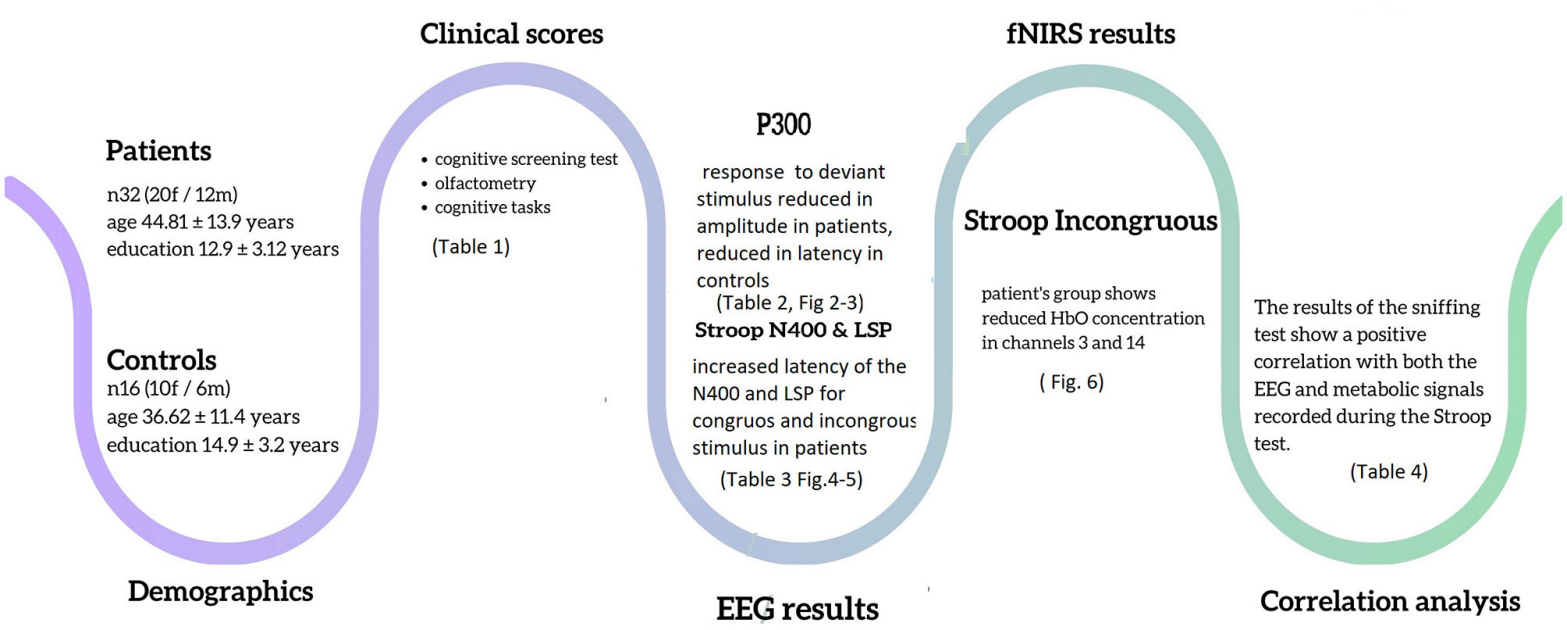
Graphical representation of the most important findings of the study.

The main results are summarized in Figure 7.

## 4. Discussion

The present study, using neuropsychological and integrated EEG/fNIRS analysis of P3 and Stroop test paradigms, revealed that subjects with mild protracted hyposmia after COVID-19 symptoms had a slight reduction in neuropsychological performances and mildly impaired event-related responses, referring to the pre-frontal regions. The Sniffin' test confirmed a light impairment of smell function, compatible with the only perceived residual symptom thereafter acute infection. These results could reinforce the hypothesis that the smell symptom could be a sign of direct viral access through olfactory mucosa, with a viral tropism against the contiguous cortical regions (Meinhardt et al., 2021).

Neuropsychological assessment in post-COVID-19 patients. Consistently with previous literature (Helms et al., 2020; Mazza et al., 2021), we observed weaker performance on some cognitive tasks in the patients' group compared to the control group. This attained the global and memory scores of the MoCA test.

The MoCA is a screening tool that objectively evaluates cognitive domains (Friedman and Miyake, 2017). The total score ranges from 0 to 30; a score of <26 indicates cognitive deficits (Nasreddine et al., 2005).

Our patients showed a mild reduction in MoCA global and memory scores, which did not correspond to a subjective impression of mental deficit. Recent studies described cognitive impairment tested with the MoCA test in patients under rehabilitation treatment who recovered after severe acute syndrome (Bek et al., 2023). We examined patients with a history of mild acute syndrome, which did not require hospitalization. The test was sensitive to their slight cognitive failure, which could be compensated by cognitive resources without causing relevant problems in everyday life.

The FAB scores were reduced in an irrelevant way as compared to no previously infected people, confirming a very light cognitive dysfunction and not influencing subjects' perception of mental trouble.

The behavioral response to the Stroop test was also delayed in post-COVID-19 patients, while we did not observe substantial errors in defining the congruence of stimuli.

The Stroop paradigm has been shown to engage PFC brain areas associated with conflict monitoring and cognitive regulation (Leung, 2000); some of the functions are outlined above. The behavioral response revealed that normal cognitive performance requests took longer time in patients with residual post-COVID-19 hyposmia, which confirms a slight prefrontal dysfunction in the cognitive domain with possible compensatory strategies.

The missing responses to the target stimuli within the P3 oddball paradigm confirmed a slight deficit and inaccuracy in task execution. The P3 paradigm tests attentional allocation and working memory (De Tommaso et al., 2020). The reaction response reflects the selection and execution processes, which were similar in time between patients and controls while missing responses could represent attention fluctuation and instability, another sign of mild frontal dysfunction (Chidharom et al., 2021).

### 4.1. P3 paradigm

In this study, we chose the three-stimulus oddball paradigm, including an infrequent, non-task-relevant stimulus within the standard and the target ones. It could disentangle a pre-attentive and unconscious response recordable over the centro-frontal regions from the conscious processing, signed by a central parietal response (De Tommaso et al., 2020). The positive P3a component is thought to be an index of automatic orienting or covert shifting of attention toward infrequent novel or salient stimuli. The frontal cortex (i.e., the orbitofrontal and inferior frontal cortices) largely contributes to the generation of such components. In accordance with the supposed frontal cortex dysfunction, we observed a selective impairment of the P3a component, both in amplitude and latency, emerging from the comparison with the control group. The P3b potential, presenting with a posterior parietal distribution, was unaffected in post-COVID-19 subjects, confirming a selective involvement of frontal-related cognitive responses.

### 4.2. Stroop task

#### 4.2.1. EEG results

In concordance with the hypothesis of mild frontal dysfunction in post-COVID-19 patients, we found a relevant delay in both the N400 and late positive potentials with normal amplitude, just confirming a sort of general bias in task execution. This delayed effect was present in post-COVID-19 subjects for both the congruent and incongruent responses, suggesting that they could have a general attentional deficit and difficulty in motor response execution rather than a specific bias in the inhibitory suppression behavior toward the wrong stimulus or a specific deficit in the semantic domain. This general delay in the requested motor response could also be a sign of mental fatigue, which was not subjectively adverted by post-COVID-19 subjects, while it is described in patients after acute infection recovery (Ceban et al., 2022). However, the frontal cortical metabolism was reduced in post-COVID-19 patients in the incongruent task, concurrent with a mild and not relevant reduction of N400 and LSP scalp distribution toward the central regions.

#### 4.2.2. fNIRS results

In the present study, fNIRS was utilized to assess the involvement of the PFC in selective attention processes and automatic response inhibition. While performing the Stroop (color-word) incongruent task, the post-COVID-19 patients showed reduced hemodynamic activations in the PFC region, on both the right and left hemispheres. The fNIRS detected metabolic changes in the 5 s following the appearance of the congruent and incongruent stimuli, thus representing the global effect of verbal stimulus categorization, right response selection, and interference suppression (Hiroyasu et al., 2011). Accordingly, the prefrontal activation during the cognitive conflict observed in control subjects was weaker in hyposmic subjects, with a possible summation of the EEG-related N400 negativity and late negativity effects.

The Stroop test was previously employed in patients recovered from acute COVID-19. Recent studies, performed 9 months after recovery from mild viral infection, found that the general performance at the Stroop test was rarely affected in non-hospitalized post-COVID-19 subjects (Kirchberger et al., 2023). Our data confirmed that subjects with previous mild viral infection and residual hyposmia could have a subtle dysfunction of cortical resources devoted to distinct executive control processes and sub-processes involved in the Stroop task without clear influence on individual functional capacities.

### 4.3. Correlation between hyposmia and cognitive tasks

Both the metabolic and EEG signals recorded during the Stroop test were positively correlated with the sniffing results, confirming that the general gap in task execution as well as the subtle deficit in cognitive interference, all attributable to frontal cortical resources, may be a consequence of viral aggression through the nasal mucosa toward the contiguous brain regions (Meinhardt et al., 2021).

## 5. Study limitations

The study was performed across two pandemic phases that occurred in south Italy (Istituto Superiore di Sanità, 2020), so the viral variant could be different in the evaluated subjects. In addition, the selected control subjects did not report previous infection or symptoms, but an asymptomatic COVID-19 form could not be excluded; it is recommended that future studies adopt a more rigorous patient selection protocol, possibly using methods such as RT-PCR (reverse transcription polymerase chain reaction) to ensure a more accurate assessment of subjects' COVID-19 history (Habibzadeh et al., 2021). The number of cases was small, as the selection criteria, based on residual smell impairment as the sole post-COVID-19 residual symptom, did not allow for finding additional cases during the study period. A further follow-up visit could just ascertain the time of persistence of smell and cognitive impairment.

## 6. Conclusion

The present study indicated that most patients who recovered from COVID-19 with persistent hyposmia 4 months after the end of the infection still presented with mild prefrontal function deficits, confirming the hypothesis about a retrograde effect of the SARS-CoV-2 virus on the brain regions contiguous to the entry zone. Recent studies confirmed the long-term persistence of both smell and cognitive dysfunction as brain fog in post-COVID-19 patients (García-Meléndez et al., 2023).

While the examined subjects had mild long COVID-19 symptoms, the presence of subtle cognitive deficits indicates the opportunity to evaluate cortical functions by means of neuropsychological and neurophysiological procedures in cases with residual hyposmia, possibly within a prospective clinical assessment.

The present study indicated that most patients who recovered from COVID-19 with persistent hyposmia 4 months after the end of the infection still presented with mild prefrontal function deficits, confirming the hypothesis about a retrograde effect of the SARS-CoV-2 virus on the brain regions contiguous to the entry zone.

While the examined subjects had mild long COVID-19 symptoms, the presence of subtle cognitive deficits indicates the opportunity to evaluate cortical functions by means of neuropsychological and neurophysiological procedures in cases with residual hyposmia, possibly within a prospective clinical assessment.

## Data availability statement

The raw data supporting the conclusions of this article will be made available by the authors, without undue reservation.

## Ethics statement

The studies involving humans were approved by Ethical Committee of Policlinico General Hospital Bari Italy. The studies were conducted in accordance with the local legislation and institutional requirements. The participants provided their written informed consent to participate in this study. Written informed consent was obtained from the individual(s) for the publication of any potentially identifiable images or data included in this article.

## Author contributions

MT: project administration, conceptualization, supervision, writing, reviewing, and editing. LC and ML: data curation, formal analysis, writing, and editing. EV and EG: data acquisition and review. AB and VB: data curation. NQ, MD, and SS: visualization and supervision. All authors contributed to the article and approved the submitted version.
